# Can nutrition-sensitive agriculture interventions address intersectional inequalities in women’s diets? A mediation analysis using cross-sectional trial data from Odisha, India

**DOI:** 10.1016/j.ajcnut.2025.05.027

**Published:** 2025-05-29

**Authors:** Emily Fivian, Helen Harris-Fry, Bhavani Shankar, Ronali Pradhan, Satyanarayan Mohanty, Shibanath Padhan, Audrey Prost, Manoj Parida, Naba K Mishra, Shibanand Rath, Suchitra Rath, Elizabeth Allen, Suneetha Kadiyala

**Affiliations:** 1 Department of Population Health, London School of Hygiene and Tropical Medicine, London, United Kingdom; 2 Department of Geography, University of Sheffield, Sheffield, United Kingdom; 3 Digital Green, New Delhi, India; 4 DCOR (Development Corner) Consulting Pvt, Ltd., Bhubaneswar, India; 5 Voluntary Association for Rural Reconstruction and Appropriate Technology (VARRAT), Kendrapara, India; 6 Institute for Global Health, University College London, London, United Kingdom; 7 Ekjut, Chakradharpur, Jharkhand, India; 8 Department of Medical Statistics, London School of Hygiene and Tropical Medicine, London, United Kingdom

**Keywords:** nutrition-sensitive agriculture, dietary diversity, equity, inequalities, India, intersectionality, participatory learning and action, maternal nutrition

## Abstract

**Background:**

Improving nutrition for all requires understanding how interventions influence nutrition inequalities within society. Intersectionality, which considers how multiple disadvantages intersect, may offer more precise insight into the equity of these interventions.

**Objectives:**

Using an intersectionality-informed approach and mediation with exposure–mediator interaction, we investigated how participation in nutrition-sensitive agriculture interventions tested in the UPAVAN trial affected inequalities in women’s diets in Odisha, India.

**Methods:**

We analyzed cross-sectional endline data from 3294 mothers of children aged 0–23 months in 111 UPAVAN intervention villages. We estimated dietary inequalities as excess relative risk of minimum dietary diversity (MDD-W) according to scheduled tribe (ST) identity (ST or non-ST), education (≥5, <5 y), or wealth (higher, lower) and comparing intersectional groups that combine ST/non-ST with education or wealth group. We used a 4-way decomposition to estimate whether these MDD-W inequalities were affected by social group differences in intervention participation rates (mediation only), participation benefits (interaction only), or both combined (mediated interaction).

**Results:**

Intervention participation and MDD-W were greater among the more advantaged groups of non-ST, higher education, or higher wealth. Often, the more disadvantaged groups had greater participation benefits (interaction only), which narrowed MDD-W inequalities. However, intersectional groups with 2 disadvantaged characteristics (e.g., poorer ST) had smaller participation benefits than those with 1 disadvantaged characteristic (e.g., wealthier ST), which widened MDD-W inequalities. Differences in participation rates had negligible effects on MDD-W inequalities. Often, any marginal widening of MDD-W inequalities due to disadvantaged groups participating less (mediation only) was suppressed by their greater participation benefits (mediated interaction).

**Conclusions:**

To our knowledge, this is the first intersectionality-informed analysis of nutrition interventions. UPAVAN interventions mostly had equitable impacts, reducing several inequalities in maternal diet quality. We demonstrate how intersectionality-informed analyses can help identify inequities in nutrition interventions and inform the design of inclusive interventions that reach and benefit the most marginalized groups.

## Introduction

The global burden of undernutrition remains disproportionately concentrated among marginalized groups [1]. India carries the greatest burden, with striking inequalities across the population [[2], [3], [4]]. For instance, half of women are anemic, but the odds are 1.3 or 1.4 times higher among the poorest or scheduled tribe (ST) (disadvantaged) groups [5]. Similarly, ∼20% of children are fed a minimally diverse diet, but the odds are ≤2-fold higher among those with more-educated mothers [6].

These trends reflect India’s distinctive social landscape shaped by factors such as caste, tribe, wealth, education, and gender [7,8]. These factors can intersect to shape nutrition inequalities, but how they do so is less well-known. Intersectionality theory captures this complexity by recognizing that individuals’ multiple social characteristics intersect within complex systems of interlocking power and oppression to shape unequal opportunities for health [9]. Applying intersectionality in research is now recognized as valuable for understanding nutrition inequalities and advancing the Sustainable Development Goals of zero hunger, reducing inequality, and leaving no one behind [[10], [11], [12]].

Rural India is a vital setting for such research as it faces the highest rates of maternal undernutrition [13] alongside multiple deep-rooted inequalities [7]. In this context, nutrition-sensitive agriculture (NSA) interventions (i.e., agriculture interventions with nutrition objectives) are now understood to be an effective way to improve maternal and child dietary quality on average [14]. However, there is sparse literature on their equity, that is, the degree to which interventions address the social, economic, or political drivers of systematic differences in nutrition outcomes [15]. NSA delivery platforms, such as extension services [16,17], mobile technologies [18], and women’s groups [[19], [20], [21], [22]], can vary in inclusivity. Structural barriers, such as high workload—which tend to affect the poorest and marginalized the most—can prevent the most nutritionally vulnerable from participating [23]. Even when included, NSA practices may not be equally accessible or feasible. For example, many require land, water, and labor, meaning that better-off groups with more resources may benefit more [24].

To our knowledge, no study has empirically examined NSA (or other nutrition) interventions from a lens of intersectionality [25]. We address this gap by unpacking the impacts of NSA interventions tested in the UPAVAN trial in rural India [20,26]. The interventions worked with women’s groups, who viewed and discussed NSA and nutrition-specific videos and used a participatory learning and action (PLA) approach. The impact evaluation found improvements in women’s and children’s dietary diversity, and per-protocol analyses suggested that intervention participation was important [26]. Using mediation with exposure–mediator interaction, we investigated whether participation rates and the benefits of participating in the interventions varied across women based on their intersecting social characteristics and, in turn, whether this affected intersectional inequalities in women’s diets.

## Methods

### UPAVAN overview

#### Study context

The UPAVAN interventions were implemented in Keonjhar, a heavily forested and landlocked district in Odisha, India. Undernutrition is widespread. Almost 70% of women are anemic [27], and ∼80% consume inadequately diverse diets [24]. Most of the population depends on subsistence farming for food and income, and almost half live below the poverty line [28]. Communities referred to as STs comprise >45% of the population [28]. These communities are considered the earliest settlers on the Indian subcontinent and were recognized as tribes during British colonial rule, then reclassified as STs in independent India [29]. STs are a heterogeneous group, with ∼700 officially recognized STs in India [30]. Their marginalization related to their indigeneity, land rights, distinct linguistic and cultural identities, and geographic isolation are well documented [31].

Other disadvantaged groups, referred to as other backward castes and scheduled castes also live in Keonjhar [24,32]. These groups are marginalized due to their caste identity and share more commonalities with mainstream Hindu society than STs [31]. Scheduled castes and STs—who have been beneficiaries of similar affirmation policies since India’s independence—are often grouped as one disadvantaged category. However, poverty reduction and political mobilization have been greater among scheduled castes [31]. Meanwhile, STs continue to face deep and persistent disadvantages, particularly in health and nutrition. They are the most undernourished in Indian society [33], even when compared with scheduled castes [3], and lag behind the national average in almost every indicator of sustainable development [34].

#### UPAVAN interventions

UPAVAN was a 4-arm cluster-randomized controlled trial carried out in 148 clusters (villages and their surrounding hamlets) in 4 blocks of Keonjhar. The UPAVAN interventions worked with women’s self-help groups, providing behavior change communication through facilitated viewings and discussions of participatory videos on NSA and nutrition-specific topics and a cycle of nutrition-specific PLA meetings [26]. Primary and secondary outcomes were the proportion of women and children consuming a minimally adequate diet (≥5 of 10 food groups for women; ≥4 of 7 food groups for children), child wasting, and maternal BMI (in kg/m^2^) [20,26].

Clusters were randomly allocated to 1 of the following 4 arms:

AGRI: Fortnightly women’s group meetings with facilitated viewings and discussions of participatory NSA videos and follow-up home visits with group participants who were pregnant or had a child aged <2 y.

AGRI-NUT: Fortnightly women’s group meetings with facilitated viewings and discussions of participatory videos, half on NSA topics and the other half on nutrition-specific topics and follow-up home visits.

AGRI-NUT+PLA: Fortnightly women’s group meetings, with half of them involving facilitated viewing and discussions of NSA videos, and the other half following a cycle of nutrition-specific PLA meetings once per month and follow-up home visits.

Control: Standard agriculture, health, and nutrition services from the government or any other organizations.

Videos were 7–15 minutes long and featured local community members discussing and demonstrating the NSA or nutrition-specific practices. Facilitators screened the videos using low-cost projectors and paused the videos at specified points to encourage discussion. Videos on NSA topics included practices aiming to increase the production of nutrient-dense foods and agricultural income, reduce costs or labor inputs, and improve women’s decision making. Videos on nutrition-specific practices focused on increasing the dietary adequacy of mothers and children.

The PLA approach incorporated into the AGRI-NUT+PLA arm involved a facilitated meeting cycle comprising the following 4 phases: *1*) group members identified and prioritized nutrition problems; *2*) group members explored causes and effects of the prioritized problems, planned local strategies to address them, decided roles and responsibilities for implementing strategies, and shared learning with the wider community; *3*) group members implemented strategies; and *4*) group members informally evaluated the process [26].

All women in intervention clusters were eligible to participate in the interventions, which were performed for 32 months between 2016 and 2019. More details of the UPAVAN interventions are found elsewhere [20,26].

### Data collection

We evaluated the impacts of the UPAVAN interventions using cross-sectional surveys at baseline (November 2016–January 2017) and endline (November 2019–January 2020) on a random sample of households with a child aged 0–23 months and a female primary caregiver aged 15–49 y. At baseline and endline, we aimed for 32 households per cluster in all 148 clusters, giving a target sample of 4736 households [26].

In this study, we used the cross-sectional endline data from 3294 mothers of children 0–23 months and their households in the 111 clusters where UPAVAN interventions were delivered [35]. Trained data collectors administered pretested questionnaires translated into Odiya language to women and their spouses (or household heads, if unavailable). Enumerators entered data using Open Data Kit software (version 1.29.3) on Android tablets. Data quality was assured by data managers doing spot-check observations on 10% and back checks (revisiting households) on 20% of all surveys. Data on dietary intake were obtained using the free recall method with standard, prespecified probes [36].

### Study variables

The variables used in this study are described in Table 1 [36]. Our study outcome is the proportion of women consuming ≥5 of 10 food groups in the previous 24 h, that is, maternal minimum dietary diversity (MDD-W)—a validated measure of micronutrient adequacy [36]. We selected this outcome based on the trial’s impact evaluation, which found improvements in dietary diversity among women and children but not women’s BMI or child wasting [26]. We focused on MDD-W to examine an outcome with a known effect, providing a foundation for subsequently exploring the intersectional equity of the impacts. This focus also addressed the scarcity of intersectionality-informed analysis of women’s nutrition in India, as existing research had mostly focused on children [25].

**TABLE 1:** Variable definitions.

Indicator	Indicator definition
Exposure and/or moderator	
Single social groups	
Non-ST/ST	Two categories—women belonging to the scheduled tribe (ST) group and women not belonging to the scheduled tribe group (non-ST) (includes scheduled caste, other backward castes, and other caste groups (often referred to as general, forward, or upper caste).
Wealth group	Two categories—a wealth score was derived as the first principal component from a principal components analysis on ownership of a range of 16 household assets, including land ownership, improved water sources, improved toilet facilities, and higher quality household dwellings. Households that fell into the top 50% were classified as higher wealth and those at the bottom as lower wealth.
Education group	Two categories—higher education, defined as women that completed lower primary school or more (≥5 y of education), and lower education, defined as those that did not (<5 y of education).
Intersectional social groups	Using the abovementioned variable definitions, we created a set of indicator variables for each of the 4 possible combinations of non-ST/ST and wealth: non-ST higher wealth, ST higher wealth, non-ST lower wealth, and ST lower wealth; and the 4 non-ST/ST and education group combinations: non-ST higher education (1), ST higher education (2), non-ST lower education (3), and ST lower education (4). We also constructed a set of indicator variables for each possible comparison between these groups, leading to 6 intersectional group comparisons for each pair of identities (between 1 and 2, 1 and 3, 1 and 4, 2 and 3, 2 and 4, and 3 and 4)
Nutritional outcome	
Minimum dietary diversity for women (MDD-W)	The proportion of women consuming ≥5 of 10 food groups in the previous 24 h. Food groups are starchy staples; beans, peas and pulses; nuts and seeds; eggs; meat and fish; dairy; dark green leafy vegetables; other vitamin A–rich fruits and vegetables; and other vegetables [36].
Mediator	
UPAVAN intervention participation	The proportion of women reporting that they attended ≥1 UPAVAN intervention video dissemination or PLA meeting in the previous 6 mo (of a maximum of 11 sessions) and being a member of a women’s self-help group.

Abbreviations: UPAVAN, Upscaling Participatory Action and Videos for Agriculture and Nutrition.

Our exposures comprised single and intersectional social groups. Given the stark and enduring disadvantage faced by STs [33], we focused on women who belong to ST communities compared with those who do not (non-ST). Statistics from UPAVAN endline survey in the control arm reinforce this focus: the proportion of women that achieved MDD-W was 42%, 41%, and 44% among scheduled castes, other backward castes, and other, respectively, but 27% among STs.

We also focused on women’s education level and household wealth, which are commonly seen as intersecting with caste and ST identity in the anthropological and sociological literature [37,38]. For education, we compared women who had ≥5 or <5 y of education (where the former indicates termination of schooling before the first cycle of mandatory education is complete). For wealth, we compared those in the top or bottom 50% of a wealth score (where wealth score is derived as the first principal component from a principal component analysis of ownership of 16 household assets). Our intersectional groups then comprised each possible combination of non-ST/ST by education or wealth group, each of which being listed in Table 1.

Our potential mediator was UPAVAN intervention participation, defined as the proportion of women who reported attending ≥1 video dissemination or PLA meeting in the last 6 months (of a maximum of 11 sessions) and being a member of a women’s self-help group.

### Analysis

We first described intervention participation (the potential mediator) within each single and intersectional social group. We then described MDD-W (the outcome) across intervention participation within these subgroups.

Next, we used state-of-the-art mediation methods, grounded in the potential outcomes framework [[39], [40], [41]], to investigate whether and how intervention participation affected social inequalities in MDD-W. The potential outcomes approach is widely recognized as more rigorous than traditional methods [42], like the approach by Baron and Kenny [43], as it defines effects using counterfactual scenarios—for example, what would have happened to inequalities in MDD-W if the groups being compared had equal levels of intervention participation?

Additionally, we applied a more advanced approach to potential outcomes-based mediation that allows for exposure–mediator interaction, which also cannot be accounted for within traditional methods [44]. Specifically, we used a novel application of VanderWeele’s 4-way decomposition [39] to unpack 3 mechanisms by which we hypothesized that the interventions may have affected intersectional inequalities in MDD-W: *1*) differences in the benefits of participating (interaction only); *2*) differences in participation rates (mediation only); and *3*) their joint contribution (mediated interaction). Distinguishing between these mechanisms is important for informing the design of more equitable interventions, as it provides maximum insight into not only whether the interventions narrowed or widened MDD-W inequalities but also how they did so. This level of insight can only be achieved through this type of mediation decomposition grounded in the potential outcomes framework [39].

**FIGURE 1:**
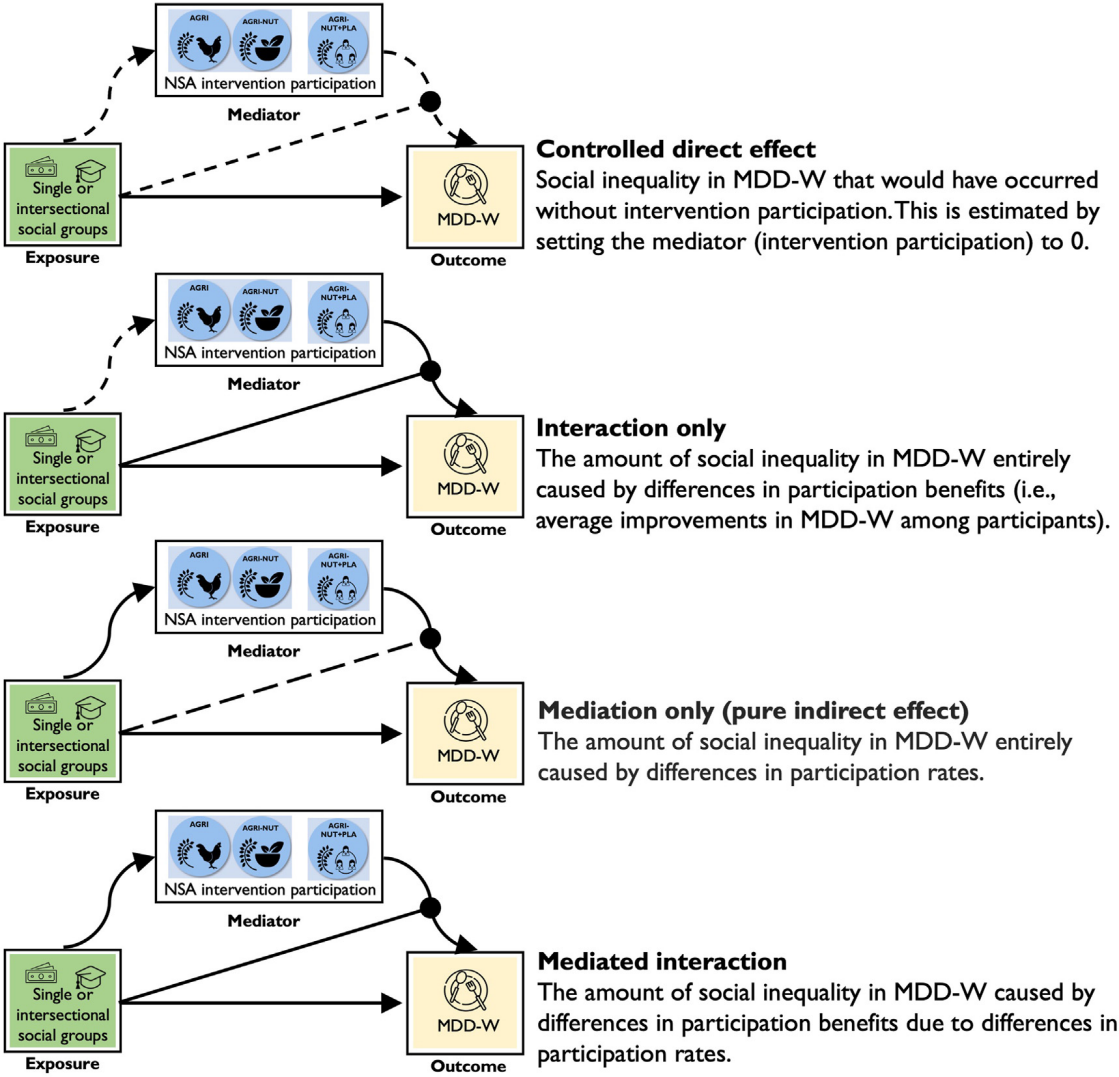
Components of the 4-way decomposition used to investigate the equity of NSA UPAVAN interventions. Notes: Solid lines indicate the path of interest; dashed lines indicate paths held constant. Arrows with circular ends indicate moderation; arrows with triangular ends indicate casual paths. MDD-W, minimum dietary diversity for women; UPAVAN, Upscaling Participatory Action and Videos for Agriculture and Nutrition.

The 4-way decomposition breaks down the observed inequalities in MDD-W between single or intersectional groups (i.e., the total effect) into 4 components [39]. Our study-specific interpretations of these components are shown in Figure 1 and described further. The mathematical expression is shown in Supplemental Methods 1**.**

#### Controlled direct effect

This refers to the social group inequality in MDD-W that would have occurred without intervention participation, that is, the amount of inequality in MDD-W that is not caused by differences in participation rates or participation benefits.

#### Interaction only

This refers to the amount of social inequality in MDD-W that is entirely caused by differences in participation benefits (i.e., average improvements in MDD-W among participants), that is, the effect of participating in the interventions on MDD-W depends on the social group, but the social group does not influence participation rates.

#### Mediation only

This refers to the amount of social inequality in MDD-W that is entirely caused by differences in participation rates, that is, the social group influences intervention participation rates but does not influence the effect of participating on MDD-W.

#### Mediated interaction

This refers to the amount of social inequality in MDD-W caused by differences in participation benefits due to differences in participation rates, that is, the effect of participating in the interventions on MDD-W depends on the social group, as in interaction only, but here, the social group also influences participation rates.

The use of the potential outcomes framework for causal interpretations requires assumptions to be defined and justified [39]. A primary assumption is that the exposure temporally precedes the mediator and that both of these precede the outcome [45]. Whether women belong to STs is determined at birth, and completion of 5 y of education is typically established in childhood. Although our wealth indicator was at greater risk of violating this assumption, our wealth score was derived from major household assets that are unlikely to change from intervention participation. Likewise, diet quality in the previous 24 h is unlikely to have influenced intervention participation. Therefore, we consider this assumption satisfied.

A second key assumption is the absence of unmeasured confounding between the exposure and mediator and between the mediator and outcome [39]. Given that our exposures are social characteristics that influence multiple interconnected aspects of life and that the interventions were designed to operate along multiple complex pathways [20], adjusting for intermediate factors could inadvertently block the mechanisms through which the social characteristics influence participation and diets. To avoid this, we rely on temporality assumptions and conceptual framing rather than statistical controls of potential confounders. This approach reflects considerations raised in intersectionality and disparity-focused causal analyses [46,47].

We conducted the 4-way decomposition using the user-written command Med4way in Stata [48]. The total effects and the 4 decomposition components were estimated using the parameter estimates from the following 2 regressions: *1*) log-binomial regressions that predict the outcome (MDD-W) as a function of the exposure (single or intersectional social group comparisons), mediator (intervention participation), and an exposure–mediator interaction term; and *2*) logistic regression models that predict the mediator as a function of the exposure.

We computed the decomposition for each single or intersectional group comparison and intervention arm. Results are crude total excess relative risk (ERR) or ERR due to each decomposition component (presented with 95% CIs). SEs are estimated from bootstrapping (1000 replications). All analyses were conducted in Stata SE/18.0.

### Analytical sample

The UPAVAN survey was not explicitly designed for intersectional analyses, and no formal power calculation was conducted for this analysis. As a result, some intersectional groups have relatively few observations. To circumvent some of this issue and improve readability, we pooled the AGRI and AGRI-NUT intervention arms but analyzed AGRI-NUT+PLA separately. We did this because the AGRI and AGRI-NUT interventions had the same model of encouraging participation through self-help groups and a similar intervention approach of viewing and discussing videos on nutrition-specific and/or NSA topics. Meanwhile, the AGRI-NUT+PLA had more community outreach activities and stronger participatory components through the cycle of PLA meetings.

As small sample sizes persisted for some comparisons and because we explored inequalities in MDD-W across the entire social spectrum of non-ST/ST by education and wealth group, formal statistical testing would carry a high risk of type I and type II errors. Hence, we focused on identifying consistent trends based on the magnitude and direction of effects rather than drawing conclusions based on statistical significance. This approach was appropriate for exploring the equity of the UPAVAN interventions, helping to assess whether different trends emerge when intersectional groups are considered when compared with single social groups, and aligned with guidance on making cautionary inferences from equity-based subgroup analyses to better understand how interventions affect health equity [49].

When reporting trends, we present the percentage point (pp) contribution of the decomposition components to the observed inequality in MDD-W. For example, if the observed inequality in MDD-W between 2 groups is an ERR of 0.80 (equivalent to a relative risk ratio of 1.80) and an ERR of 0.13 due to interaction only (or mediation only or mediated interaction), then we can say that it explains 13 pp of the observed inequality and implies a widening of the observed inequality in MDD-W. In contrast, an ERR of −0.13 implies a narrowing of the observed inequality in MDD-W by 13 pp.

### Ethics

Ethical approval was granted from the Odisha Government’s Institutional Review Board, Research and Ethics Committee, Department of Health and Family Welfare, Government of Odisha (date approved 3 September, 2016, letter number 141/SHRMU) and from the London School of Hygiene and Tropical Medicine (LSHTM) Interventions Research Ethics Committee (date approved 10 October, 2016, reference number 11 357; trial registration: ISRCTN65922679). We obtained written informed consent for participation in interviews and the use of pseudonymized data from participants. For mothers 15–17 y old, we obtained assent from the mothers and informed written consent from a representative adult (e.g., their spouse or in-laws).

## Results

The participant flow chart is shown in Supplemental Figure 1, and respondent characteristics are given in Table 2. Most respondents had <2.5 acres of land, 70% had ≥5 y of education, and ∼ 60% belonged to the ST group. Of the non-ST group, 20%–25% were from scheduled castes, 62%–74% from other backward castes, and 6%–12% from other castes (sometimes referred to as general or upper caste). We had small sample sizes for the non-ST group with lower education or lower wealth, reflecting relatively higher educational and economic outcomes among the non-ST group. Among intervention participants, the mean number of video disseminations or PLA meetings attended in the previous 6 months was 7.3 in the AGRI and AGRI-NUT arms and 6.6 in the AGRI-NUT+PLA arm, of a maximum of 11 sessions.

**TABLE 2:** Participant characteristics.

Characteristic	AGRI and AGRI-NUT (*n* = 2155)		AGRI-NUT+PLA (*n* = 1139)	
	*n*	Mean (SD) or *n* (%)	*n*	Mean (SD) or *n* (%)
Woman’s age (y), mean (SD)	2155	24.6 (4.3)	1139	24.8 (4.4)
Size of landholding	2149		1136	
<2.5 acres		1729 (80.0)		904 (79.6)
≥2.5 acres		429 (20.0)		232 (20.4)
Non-ST/ST group	2153		1138	
ST		1329 (61.7)		662 (58.2)
Non-ST		824 (38.3)		476 (41.8)
Caste of non-ST group	824		476	
Scheduled caste		167 (20.3)		121 (25.4)
Other backward caste		610 (74.0)		299 (62.3)
Other caste		47 (5.7)		56 (11.8)
Education in years, mean (SD)	2155	6.8 (4.4)	1139	6.9 (4.6)
Education category	2155		1139	
Lower education (<5 y)		656 (30.4)		341 (29.9)
Higher education (≥5 y)		1499 (69.6)		798 (70.1)
Wealth group	2153		1138	
Lower wealth		1095 (50.9)		555 (48.9)
Higher wealth		1058 (49.1)		583 (51.2)
Non-ST/ST and education	2153		1138	
ST with lower education		541 (25.1)		291 (25.6)
ST with higher education		788 (36.6)		371 (32.6)
Non-ST with lower education		114 (5.3)		50 (4.4)
Non-ST with higher education		710 (33.0)		426 (37.4)
Non-ST/ST and wealth	2153		1138	
ST with lower wealth		834 (38.7)		425 (37.4)
ST with higher wealth		495 (23.0)		237 (20.8)
Non-ST with lower wealth		261 (12.1)		130 (11.4)
Non-ST with higher wealth		563 (26.2)		346 (30.4)
UPAVAN intervention participation	2155	647 (30.0)	1139	341 (29.9)
Number of video viewings or PLA meetings attended in past 6 mo among intervention participants (range, 1–11), mean (SD)	657	7.3 (3.4)	341	6.6 (3.0)
Minimum dietary diversity for women (MDD-W)	2155	798 (37.0)	1139	429 (42.1)

Notes: Values are *n* (%) unless specified. AGRI and AGRI-NUT, interventions with women’s groups using participatory videos on nutrition-sensitive agriculture and nutrition-specific topics; AGRI-NUT+PLA, same as AGRI and AGRI-NUT plus nutrition-specific participatory learning and action (PLA) meetings; non-ST, women not from scheduled tribes; ST, women from scheduled tribes; UPAVAN, Upscaling Participatory Action and Videos for Agriculture and Nutrition. Higher and lower wealth is defined as being in the top or bottom 50% of a wealth score derived as the first principal component from a principal component analysis of ownership of 16 household assets. Study variables with incomplete observations (non-ST/ST group and wealth group; 0.09%) are due to missing responses in the male survey, where these data were collected.

### Descriptive results of intervention participation and MDD-W

Table 3 shows intervention participation rates across the single and intersectional social groups, and MDD-W by participation status, within these subgroups. Participation rates ranged from 21% to 36% and were generally higher among more advantaged women (higher education, higher wealth, or non-ST groups). Participation rates were greater among all higher-educated women (30%–36%) than those among lower-education women (21%–28%) in non-ST and ST groups. There was slightly less variation in participation rates across non-ST/ST by wealth groups. Still, rates were the lowest among the most disadvantaged intersectional group of poorer ST women.

**TABLE 3:** Participation rates in nutrition-sensitive agriculture interventions and MDD-W by intervention participation.

	AGRI and AGRI-NUT			AGRI-NUT+PLA		
	Intervention participation, *n*/*N* (%)	MDD-W		Intervention participation, *n*/*N* (%)	MDD-W	
		Nonparticipants (%)	Participants (%)		Nonparticipants (%)	Participants (%)
Non-ST/ST group						
ST	361/1329 (27.2)	29.2	42.4	182/662 (27.5)	33.8	44.5
Non-ST	286/824 (34.7)	42.8	45.5	159/476 (33.4)	47.3	54.1
Education group						
Lower education	154/656 (23.5)	21.1	27.9	75/341 (22.0)	24.1	32.0
Higher education	493/1499 (32.9)	40.7	48.7	266/798 (33.3)	46.6	53.8
Wealth group						
Lower wealth	301/1095 (27.5)	25.7	36.2	153/555 (27.6)	26.9	43.1
Higher wealth	346/1058 (32.7)	43.4	50.3	188/583 (32.3)	51.7	53.7
Non-ST/ST and education						
ST with lower education	124/541 (22.9)	18.9	23.4	61/291 (21.0)	23.5	29.5
ST with higher education	237/788 (30.1)	37.0	52.3	121/371 (32.6)	43.2	52.1
Non-ST with lower education	30/144 (20.8)	31.0	46.7	14/50 (28.0)	27.8	42.9
Non-ST and higher education	256/710 (36.1)	44.9	45.3	145/426 (34.0)	49.8	55.2
Non-ST/ST and wealth						
ST with lower wealth	218/834 (26.1)	25.2	34.9	108/425 (25.4)	26.8	38.0
ST with higher wealth	143/495 (28.9)	36.4	53.9	74/237 (31.2)	47.2	54.1
Non-ST with lower wealth	83/261 (31.8)	27.5	39.8	45/130 (34.6)	27.1	55.6
Non-ST with higher wealth	203/563 (36.1)	50.3	47.8	114/346 (32.9)	54.7	53.5

Notes: AGRI and AGRI-NUT, interventions with women’s groups using participatory videos on nutrition-sensitive agriculture and nutrition-specific topics; AGRI-NUT+PLA, same as AGRI and AGRI-NUT plus nutrition-specific participatory learning and action meetings; MDD-W, minimum dietary diversity for women; non-ST, women not from scheduled tribes; ST, women from scheduled tribes. Higher and lower education is defined as women with ≥5 or <5 y of schooling; higher and lower wealth is defined as being in the top or bottom 50% of a wealth score derived as the first principal component from a principal component analysis of ownership of 16 household assets.

The proportion of women who achieved MDD-W was consistently greater among participants than that among nonparticipants, with an exception: the most advantaged intersectional group of wealthier non-ST women.

### Four-way decomposition results: intersectional inequalities in MDD-W decomposed by intervention participation

**FIGURE 2:**
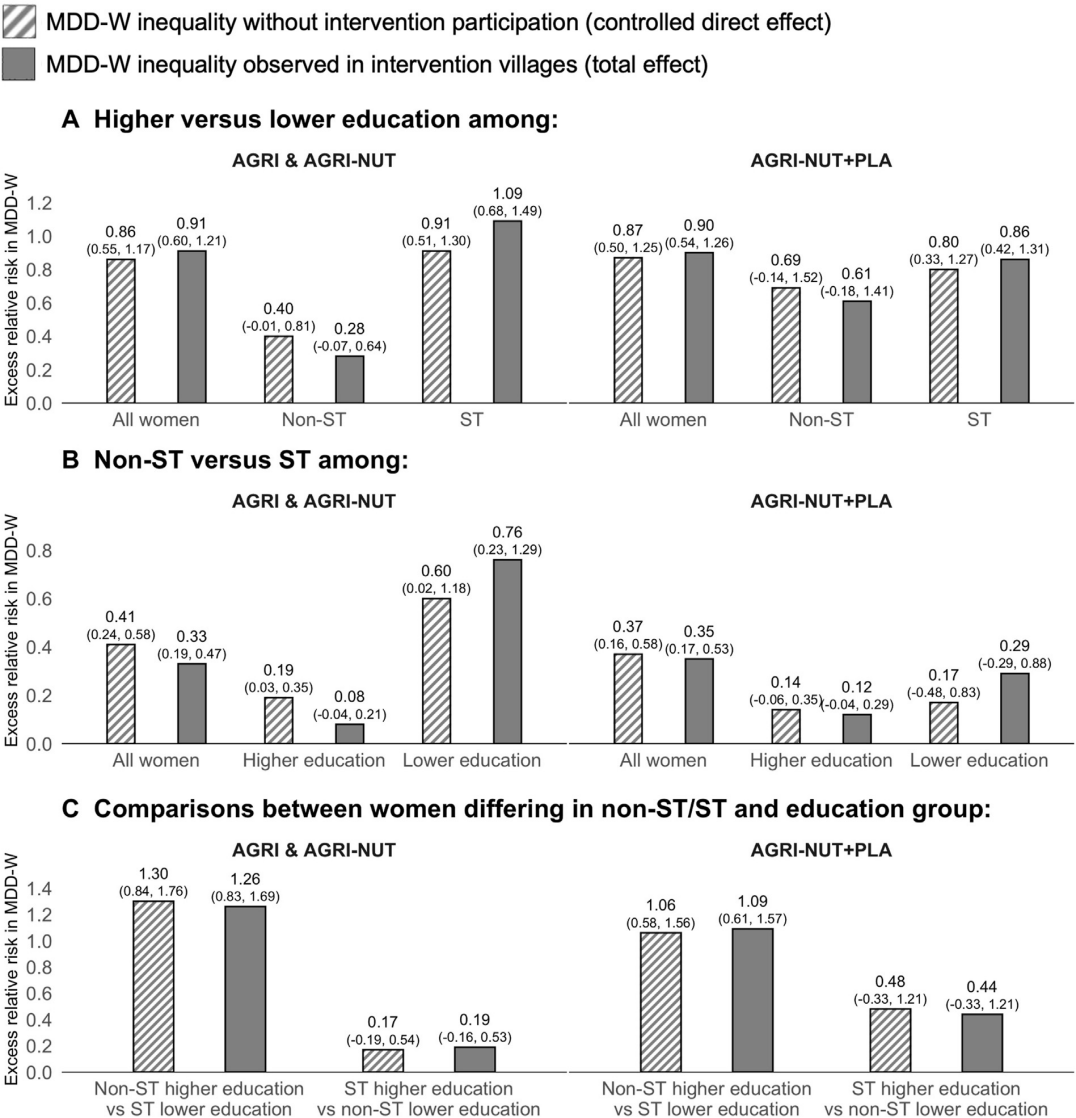
Decomposition of non-ST/ST and educational inequalities in MDD-W by participation in nutrition-sensitive agriculture interventions: total and controlled direct effects. Notes: Dashed bars, MDD-W inequality that would have occurred without intervention participated (controlled direct effect); solid bars, MDD-W inequality observed in intervention villages (total effect). (A) MDD-W compared between higher and lower education groups among all women and by ST status; (B) MDD-W compared between non-ST and ST groups among all women and by education group; (C) MDD-W compared between women differing in non-ST/ST and education groups. Results are from 4-way decomposition analyses. CIs shown in brackets above bars are normal-based and calculated from bootstrapped standard errors (1000 replications). AGRI and AGRI-NUT, interventions with women’s groups using participatory videos on nutrition-sensitive agriculture and nutrition-specific topics; AGRI-NUT+PLA, same as AGRI and AGRI-NUT plus nutrition-specific participatory learning and action meetings; MDD-W, minimum dietary diversity for women; non-ST, women not from scheduled tribes; ST, women from scheduled tribes. Higher and lower education is defined as women with ≥5 or <5 y of schooling. Sample sizes (left to right within each panel): (A) 2155, 824, 1329, 1139, 476, and 662; (B) 2153, 1498, 655, 1138, 797, and 341; (C) 1251, 902, 717, and 421.

**FIGURE 3:**
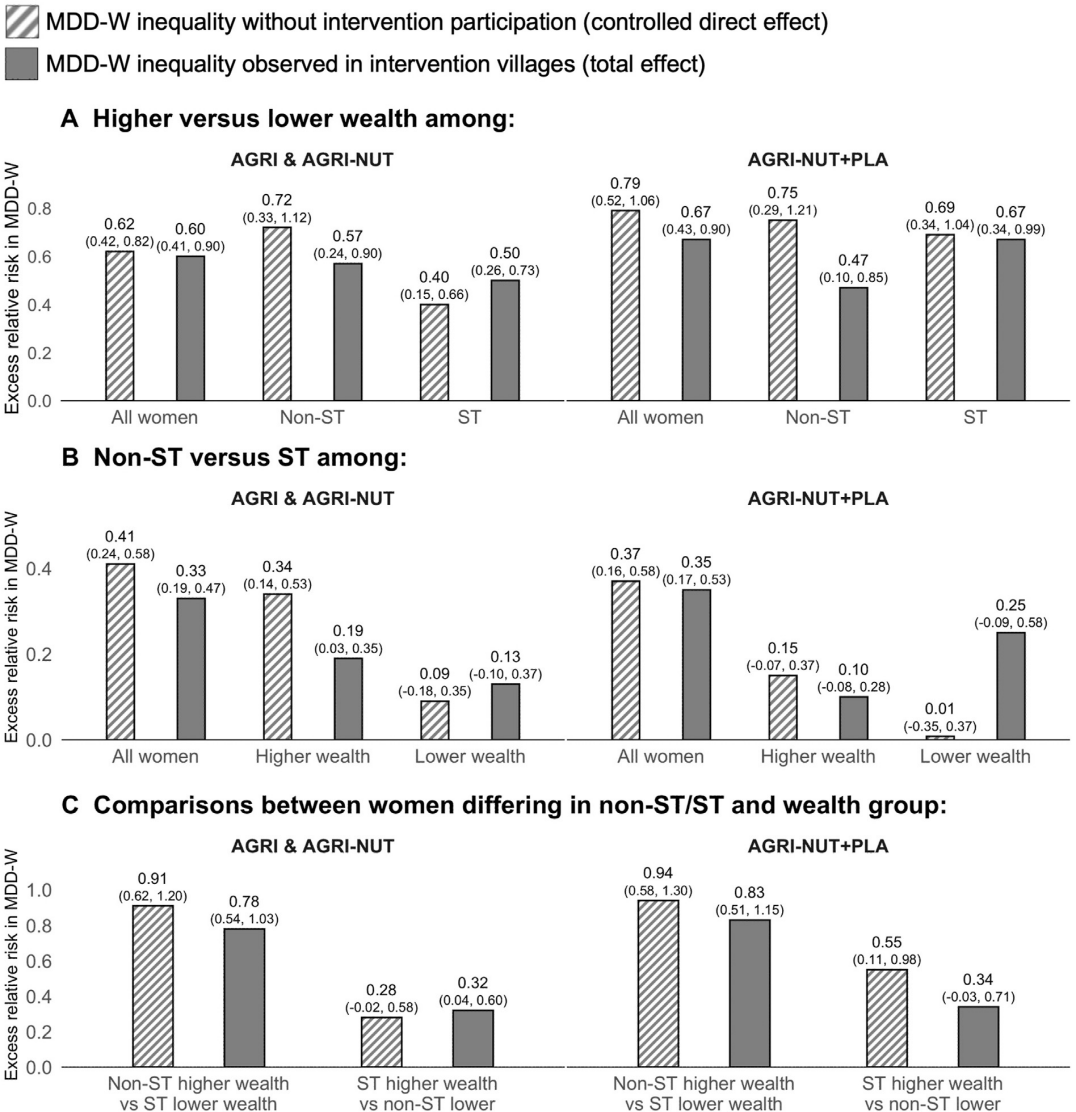
Decomposition of non-ST/ST and wealth inequalities in MDD-W by participation in nutrition-sensitive agriculture interventions: total and controlled direct effects. Notes: Dashed bars, MDD-W inequality that would have occurred without intervention participation (controlled direct effect); solid bars, MDD-W inequality observed in intervention villages (total effect). (A) MDD-W compared between higher and lower wealth groups among all women and by ST status; (B) MDD-W compared between non-ST and ST groups among all women and by wealth group; (C) MDD-W compared between women differing in non-ST/ST and wealth groups. Results are from a 4-way decomposition analysis. CIs shown in brackets above bars are normal-based and calculated from bootstrapped standard errors (1000 replications). AGRI and AGRI-NUT, interventions with women’s groups using participatory videos on nutrition-sensitive agriculture and nutrition-specific topics; AGRI-NUT+PLA, same as AGRI and AGRI-NUT plus nutrition-specific participatory learning and action meetings; MDD-W, minimum dietary diversity for women; non-ST, women not from scheduled tribes; ST, women from scheduled tribes. Higher and lower wealth is defined as being in the top or bottom 50% of a wealth score derived as the first principal component from a principal component analysis of ownership of 16 household assets. Sample sizes (left to right within each panel): (A) 2153, 824, 1329, 1138, 476, and 662; (B) 2153, 1058, 1095, 1138, 583, and 555; (C) 1392; 756, 771, and 367.

This section presents the 4-way decomposition analysis results, unpacking whether intersectional inequalities in MDD-W were affected by intervention participation. We report results for non-ST/ST and education and non-ST/ST and wealth together, as we found consistent trends. We first explored inequalities in MDD-W that would have occurred without intervention participation (controlled direct effects). We then assessed whether inequalities in MDD-W differ in magnitude from what was observed in the intervention villages (total effects). The results for non-ST/ST and education are shown in Figure 2, and the results for non-ST/ST and wealth are shown in Figure 3.

#### Inequalities in MDD-W without intervention participation

When looking at what would have occurred without intervention participation (dashed bars in FIGURE 2, FIGURE 3), we found that inequalities in MDD-W were substantial. These inequalities follow the expected trend, with a greater proportion of women achieving MDD-W among more advantaged groups. For instance, Figure 2A shows the proportion achieving MDD-W was >80% greater among higher-educated women than that among lower-educated women (AGRI and AGRI-NUT—ERR: 0.86; 95% CI: 0.55, 1.17; AGRI-NUT+PLA—ERR: 0.87; 95% CI: 0.50, 1.25) and that educational inequality in MDD-W persisted within non-ST/ST intersections. The same pattern held for wealth inequality in MDD-W (Figure 3A).

Figure 2B shows that the proportion of women achieving MDD-W was ∼40% greater for non-ST than ST women (AGRI and AGRI-NUT—ERR: 0.41; 95% CI: 0.24, 0.58; AGRI-NUT+PLA—ERR: 0.37; 95% CI: 0.16, 0.58). Although some trends suggested that non-ST/ST inequality in MDD-W persisted within intersections of wealth and education, there was no evidence of non-ST/ST inequality in MDD-W among poorer women (FIGURE 2, FIGURE 3B).

The starkest inequality in MDD-W was found when comparing the least and most disadvantaged intersectional groups. For instance, the proportion achieving MDD-W was >100% greater among higher-educated non-ST than that among lower-educated ST women (AGRI and AGRI-NUT—ERR: 1.30; 95% CI: 0.84, 1.76; AGRI-NUT+PLA—ERR: 1.06; 95% CI: 0.58, 1.56) (Figure 2C). As a robustness check, we compared these controlled direct effects with observed inequalities in MDD-W in UPAVAN control villages and found the same trends.

#### Contrasting inequalities in MDD-W with and without intervention participation

In several cases, the observed inequalities in MDD-W (solid bars of FIGURE 2, FIGURE 3) differed in magnitude from what would have occurred without intervention participation, suggesting that the interventions influenced inequalities in MDD-W. Where differences occurred, we observed the following trend: in most cases, the observed inequalities in MDD-W between single and intersectional groups appeared narrower than what would have occurred without intervention participation. However, we observed the opposite when comparing the middle intersectional groups (i.e., those with 1 disadvantaged characteristic) with the most disadvantaged intersectional groups (i.e., those with 2 disadvantaged characteristics), where observed inequalities in MDD-W appeared wider.

#### The role of the intervention participation in inequalities in MDD-W: interaction, mediation, or both?

**FIGURE 4:**
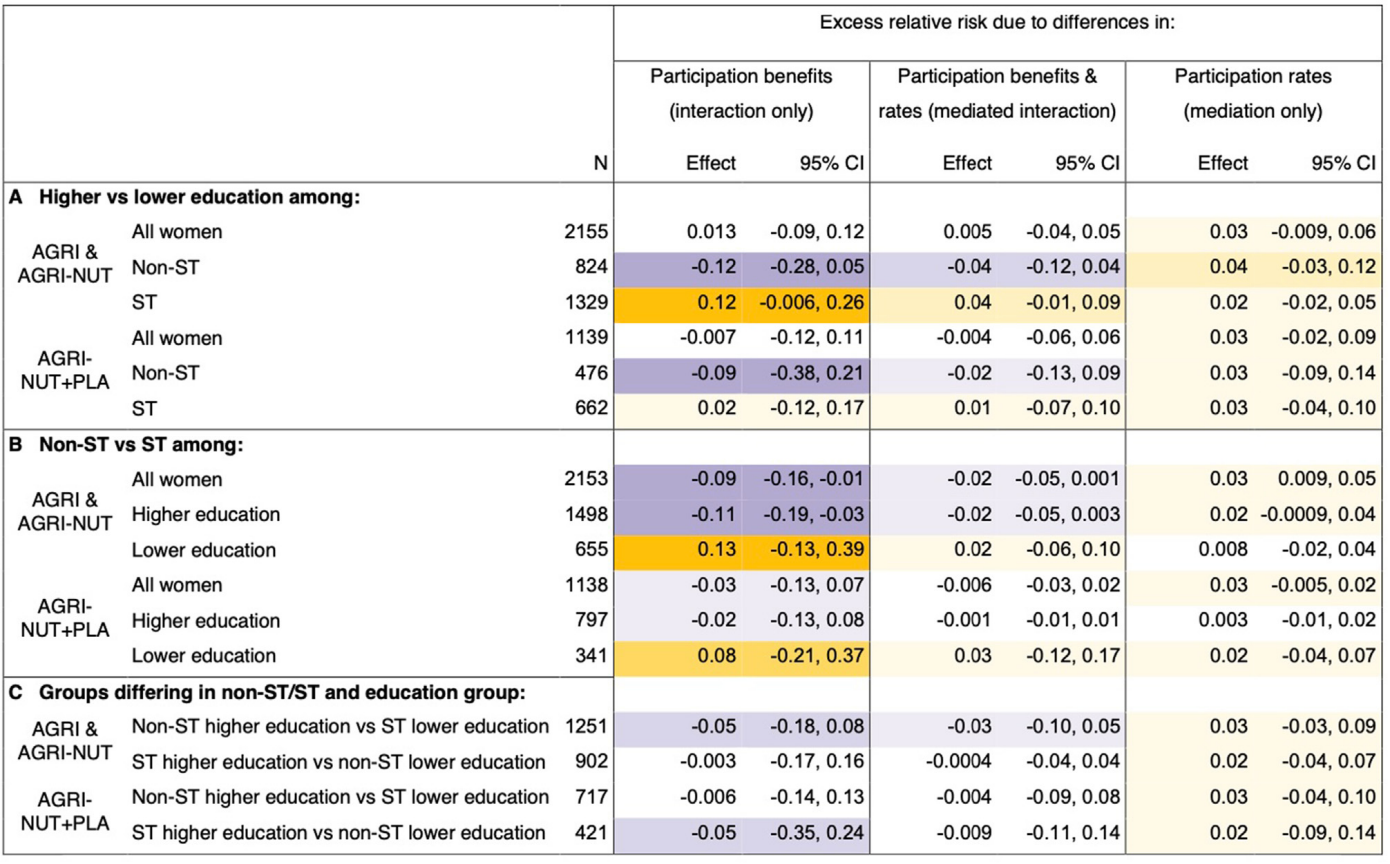
Decomposition of non-ST/ST and educational inequalities in MDD-W by participation in nutrition-sensitive agriculture interventions: interaction, mediation or both? Notes: Purple shading corresponds to effects that narrowed inequalities in MDD-W, and orange shading corresponds to effects that widened them. Darker shades indicate greater effect sizes. Results are from 4-way decomposition analyses. CIs are normal-based and calculated from bootstrapped standard errors (1000 replications). AGRI and AGRI-NUT, interventions with women’s groups using participatory videos on nutrition-sensitive agriculture and nutrition-specific topics; AGRI-NUT+PLA, same as AGRI and AGRI-NUT plus nutrition-specific participatory learning and action meetings; MDD-W, minimum dietary diversity for women; non-ST, women not from scheduled tribes; ST, women from scheduled tribes. Higher and lower education is defined as women with ≥5 or <5 y of schooling.

**FIGURE 5:**
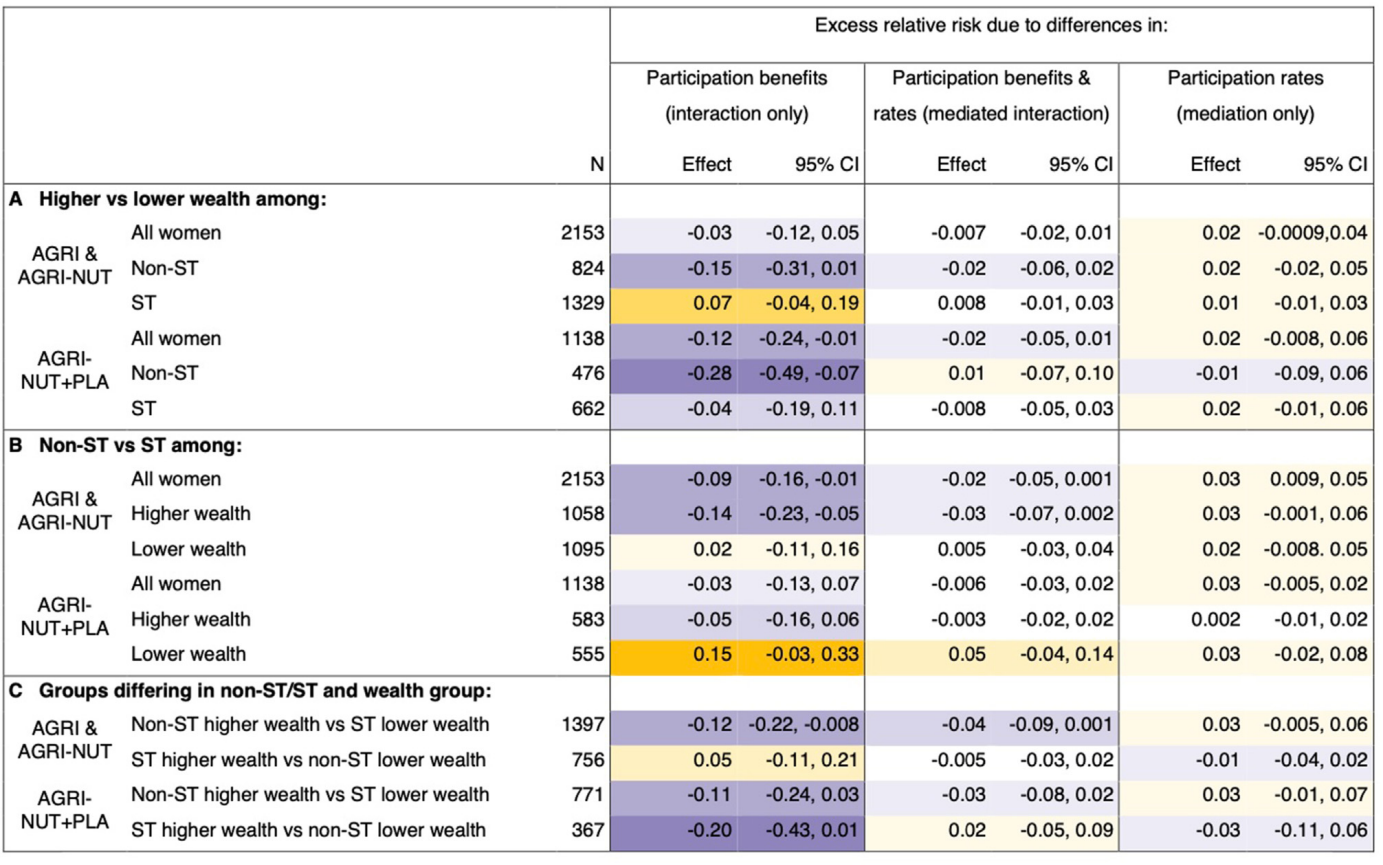
Decomposition of non-ST/ST and wealth inequalities in MDD-W by participation in nutrition-sensitive agriculture interventions: interaction, mediation or both? Notes: Purple shading corresponds to effects that narrowed inequalities in MDD-W, and orange shading corresponds to effects that widened them. Darker shades indicate greater effect sizes. Results are from 4-way decomposition analyses. CIs are normal-based and calculated from bootstrapped standard errors (1000 replications). AGRI and AGRI-NUT, interventions with women’s groups using participatory videos on nutrition-sensitive agriculture and nutrition-specific topics; AGRI-NUT+PLA, same as AGRI and AGRI-NUT plus nutrition-specific participatory learning and action meetings; MDD-W, minimum dietary diversity for women; non-ST, women not from scheduled tribes; ST, women from scheduled tribes. Higher and lower wealth is defined as being in the top or bottom 50% of a wealth score derived as the first principal component from a principal component analysis of ownership of 16 household assets.

Next, we investigated which remaining decomposition components explained any differences between the observed inequalities in MDD-W and what would have occurred without intervention participation. FIGURE 4, FIGURE 5 show the results for the remaining 3 decomposition components for non-ST/ST and education and non-ST/ST and wealth, respectively.

##### Differences in participation benefits (interaction only)

Results in the interaction only columns suggest that there were differences in the benefits of participating in the interventions and that this affected several inequalities in MDD-W. Where this occurred, differences in participation benefits often narrowed inequalities in MDD-W, as the more disadvantaged groups benefited more. However, the opposite pattern was observed when comparing middle intersectional groups with the most disadvantaged—that is, poorer or less-educated non-ST compared with ST women; and wealthier or more-educated ST women compared with poorer or less-educated ST women.

We used non-ST/ST inequalities in MDD-W among all women and within education intersections to illustrate these trends (Figure 4B). Greater intervention benefits among ST than those among non-ST women narrowed non-ST/ST inequalities in MDD-W by 9 pp among all women and 11 pp among higher-educated women (AGRI and AGRI-NUT only). However, the opposite occurred among lower-educated women, where smaller intervention benefits among ST women widened non-ST/ST inequalities in MDD-W by 13 pp in AGRI and AGRI-NUT and 8 pp in AGRI-NUT+PLA.

Despite the most disadvantaged sometimes benefiting less than middle intersectional groups, trends suggest the most disadvantaged still benefited from participating. This is shown through greater participation benefits among poorer ST (most disadvantaged) than wealthier non-ST women (least disadvantaged), narrowing inequalities in MDD-W by ≤12 pp (Figure 5C).

Further, some trends suggest that the AGRI-NUT+PLA approach may have been more equitable in terms of benefits to poorer and less-educated women. For example, although differences in intervention benefits appeared to widen education inequality in MDD-W among ST women in AGRI and AGRI-NUT, there was no suggestion of this in AGRI-NUT+PLA (Figure 4A). Similarly, differences in intervention benefits appeared to reduce wealth inequality in MDD-W to a greater extent and more consistently across different subgroups in AGRI-NUT+PLA (Figure 5A, C).

##### Differences in participation rates and benefits (mediation and mediated interaction)

On the contrary, differences in participation rates did not meaningfully affect inequalities in MDD-W. Looking first at the contribution of differences in participation alone (mediation-only column in FIGURE 4, FIGURE 5), we found that almost all effects operate toward widening MDD-W inequalities, as expected from descriptive results indicating greater participation rates among more advantaged groups. However, effects are consistently small, with MDD-W inequalities widening by a maximum of 4 pp. This suggests that participation gaps were likely too small to meaningfully affect inequalities in MDD-W.

Similarly, the effects of intervention participation on MDD-W inequalities that were due to mediated interaction (the differences in participation benefits that were due to differences in participation rates) were also consistently small and nonmeaningful. In many cases, a potential widening (albeit very small) of inequality in MDD-W due to greater participation rates among more advantaged groups (mediation only) were suppressed by greater participation benefits among more disadvantaged groups (mediated interaction). For instance, looking at education inequality in MDD-W among non-ST women in AGRI and AGRI-NUT (Figure 4A), greater participation rates among higher-educated non-ST women widened MDD-W inequality by 4 pp. However, because lower-educated non-ST women benefited more from their participation, the mediated interaction shows a narrowing of education inequality in MDD-W by 4 pp, effectively cancelling out the mediation effect.

#### Inequalities in MDD-W across various intervention participation rates

In Supplemental Figures 2–4, we visually demonstrate the role of differences in participation benefits on inequalities in MDD-W by plotting the controlled direct effect when participation rates are fixed to 0% (as has been done so far), 25%, 50%, 75% and 100%. Where participation benefits were greater among more disadvantaged groups, the plots show how these inequalities in MDD-W would further narrow at higher participation rates. The opposite occurs where participation benefits were greater among the more advantaged groups. These plots also help visualize the importance of an intersectional approach. For example, in AGRI and AGRI-NUT (left panels), as participation rates increase, education inequalities in MDD-W overall remain constant (Supplemental Figure 2D), narrow among the non-ST group (Supplemental Figure 2E), but widen among the ST group (Supplemental Figure 2F).

## Discussion

We used an intersectionality-informed approach to examine how NSA interventions tested in the UPAVAN trial affected intersectional inequalities in women’s diet quality in rural Odisha. First, we found prominent inequalities in MDD-W, with lower-educated, poorer, and ST women at greater risk of dietary inadequacy. These inequalities were amplified when women faced multiple disadvantages. For example, over twice as many higher-educated non-ST women achieved MDD-W than lower-educated ST women.

Second, we found that these MDD-W inequalities were affected by differences in the extent to which women benefited from participating in the interventions. Where differences occurred, trends suggested that middle intersectional groups (i.e., those with 1 disadvantaged characteristic) benefited the most, followed by the most disadvantaged (i.e., those with 2 disadvantaged characteristics), and then the least disadvantaged (i.e., those with 2 advantaged characteristics). As a result, differences in participation benefits generally narrowed MDD-W inequalities, except when comparing middle intersectional groups with the most disadvantaged, where greater benefits among middle intersectional groups widened MDD-W inequalities. Our intersectionality-informed approach was necessary for revealing this trend. Finally, despite greater participation rates among more advantaged women, this had negligible impacts on MDD-W inequalities.

Our findings concerning prominent social inequalities in women’s diet in rural, disadvantaged communities reinforce the need for nutrition actions that improve diet quality on average and reduce social inequalities within them. The UPAVAN impact evaluation [26], and our study indicate progress toward these objectives. This was likely attributable to UPAVAN’s focus on promoting practices that required few resources and locally feasible solutions, and responding to constraints faced by poor and vulnerable households [50,51]. Other participatory women’s group interventions have had equitable impacts. An intervention in Odisha and Jharkhand found greater reductions in neonatal mortality rates among the most than those among the least marginalized groups [52]. Potential explanatory mechanisms included high intervention uptake among marginalized groups, inclusive behavior change communication strategies, and having intervention facilitators from STs [52].

Consistent with intersectionality theory, our findings demonstrated how multiple disadvantages can be compounded [53]. This was evident in the smaller participation benefits among those with 2 disadvantaged characteristics compared with those with 1. There are several possible explanations for this. First, the study population, on average, is multidimensionally poor. Therefore, additional efforts beyond NSA, such as improving the reach and utilization of social safety nets and other welfare programs [54], are likely needed to achieve equitable impacts across the scale. In the longer term, investments in nutrition-sensitive actions that reduce structural vulnerability through improvements in education and household wealth will also be critical for sustained and equitable improvements in nutrition [55].

Second, the most disadvantaged women likely faced greater resource constraints. This aligns with the UPAVAN process evaluation, which showed that women facing multiple constraints (such as limited land, water, and low family support) felt less able to adopt the promoted practices [50]. Nonresource constraints may also play a role. For instance, lower education can restrict personal agency, which may affect confidence in meetings and motivation to adopt behaviors [56]. This may have been exacerbated by ST identity, which can also limit confidence due to well-documented discrimination against ST women [30]. A qualitative investigation of an NSA intervention delivered through self-help groups in Jharkhand supports these explanations [57]. They showed that low education or marginalized caste status made women feel less confident to approach other group members, receive help from implementation staff, or actively participate in discussions [57].

Although we found that participation rates were greater among more advantaged women, participation gaps were likely too small to meaningfully impact MDD-W inequalities. In several cases, any small widening of MDD-W inequalities from this was suppressed by greater participation benefits among the more disadvantaged women who did participate. Despite this, establishing strategies to increase participation inclusively would further improve diets and narrow several inequalities within them. Participation barriers found in the UPAVAN process evaluation included a lack of interest in participating (due to the belief they could not adopt the promoted practices) and long travel times to meetings [50]. Another study found that lack of family support was a barrier to participation in a health intervention in rural India, and women who had low education were more likely to mention a lack of family support [58]. Given that lack of family support was also a barrier to adopting practices in UPAVAN [50], interventions that include whole families may enhance NSA intervention effectiveness and equity by strengthening inclusion and pathways to impact [59].

Some of our findings suggest that NSA interventions with PLA may have more equitable impacts. This could be explained by the process involved in the PLA cycle, where groups prioritize problems and collectively identify and implement solutions with their communities [60]. As such, the nutrition problems addressed, and strategies implemented were perhaps more relevant and feasible for vulnerable groups. Other studies have also shown PLA to be inclusive and beneficial for poorer, more marginalized groups [52,61,62]. There is evidence that PLA is also cost-effective [60,63], including the economic evaluation of the UPAVAN trial [64].

### Strengths and limitations

Our study has several strengths. To our knowledge, this is the first study to use an intersectionality-informed approach to empirically examine the impacts of nutrition interventions [25], offering novel insight into nutrition intervention equity. We also demonstrated a novel methodological contribution by applying casual mediation with exposure–mediator interaction. This approach allows us to advance beyond assessing whether interventions affect inequalities in outcomes, to exploring how it does so. In doing so, we provide more actionable insights for future intervention design.

We note the following limitations. First, the cross-sectional design increases the risk of bias in our estimates. Additionally, we did not adjust for potential confounders among social characteristics, participation, and diet quality, as many such variables may lie on the causal pathways. However, we acknowledge the possibility of unmeasured confounding in the mediator–outcome relationship, which could also bias our estimates. For instance, individual motivation may influence both participation and diet quality, acting as a confounder. Yet, if motivation is socially patterned, then adjusting for it would risk obscuring the very inequalities we aimed to capture. Second, our trial data were not originally designed for intersectionality-informed analyses. Like most intersectionality studies, our analysis is likely underpowered and could have included multiple statistical tests, which would have carried a high risk of type I and II errors. To avoid this, we relied on overall trends to determine meaningful results. To our knowledge, no public health trial has been explicitly designed for such analyses, yet these analyses are crucial for hypothesis generation and designing more inclusive interventions [65]. Finally, we note that pooling multiple castes within the non-ST group may cause differences between them to be overlooked. Nonetheless, we believe the social grouping chosen best balances analytical feasibility, conceptual relevance, and socioeconomic realities.

## Conclusion

We demonstrate how intersectionality-informed analyses can help to identify inequities in nutrition interventions, which can support the design of inclusive interventions and policy strategies. We also demonstrate how novel casual methods can unpack crucial questions about intervention equity that are difficult to answer through trial design alone. The UPAVAN interventions showed promise for reducing intersectional inequalities in dietary outcomes. Scaling up such interventions, alongside targeted strategies across other sectors, will be imperative for achieving global goals to eliminate hunger for all.

## Author contributions

The authors’ responsibilities were as follows – EF, HH-F, BS, EA, SK: designed the research; EF, RP, SM, SP, AP, NKM, ShR, SuR, SK: conducted the research; EF: analyzed data and performed statistical analysis; EF, HH-F, BS, SK: wrote the article; EF: had primary responsibility for final content; and all authors: have read and approved the final manuscript.

## Data availability

Data described in the manuscript and codebook is available from LSHTM Data Compass: Upscaling Participatory Action and Videos for Agriculture and Nutrition (UPAVAN) study data, https://doi.org/10.17037/DATA.00003642. This research article contains the underlying data: UPAVAN_mother_anthro_indicators (restricted access). Because the participant information sheet did not specify that the data would be made open-access in a public repository, access to the data will only be granted once users have agreed to a data-sharing agreement and provided written plans and justification for what is proposed with data. Ethical approval may be required. Data access may be obtained by submitting a request to the LSHTM data repository. The analytic code is publicly and freely available on GitHub: https://github.com/emilycfivian/Intersectionality-in-NSA-interventions-Analytical-code.

## Funding

Funding of EF, BS, and SK was provided by Innovative Methods and Metrics for Agriculture and Nutrition Actions (IMMANA), which is cofunded by the UK Foreign and Commonwealth Development Office (FCDO) [Project 300654] and the Gates Foundation [INV-002962/OPP1211308]. Funding of EF, HH-F, SK, and BS was provided by the INFUSION Planning Grant funded by the Gates Foundation [Investment ID: INV-036930]. Funding of HH-F was provided by a Sir Henry Wellcome grant (210894/Z/18/Z). The UPAVAN trial was funded by the Gates Foundation and UK Aid from the UK Government [OPP1136656]. Substantial co-funding of the UPAVAN trial was also provided by the USAID-funded project Digital Integration to Scale Gender-Sensitive Nutrition Social and Behavior Change Communication, implemented by Digital Green [Cooperative Agreement No. AID-386-A-15–00008]. The funders of this work had no role in study design, implementation, data collection, analysis, interpretation or writing of the article.

## Conflicts of interest

The authors report no conflicts of interest.
