# Supplementary material for: Can nutrition-sensitive agriculture interventions address intersectional inequalities in women’s diets? A mediation analysis using cross-sectional trial data from Odisha, India
Source: Am J Clin Nutr. 2025 May 29;122(2):460–73. doi: 10.1016/j.ajcnut.2025.05.027 (PMC12405785; doi:10.1016/j.ajcnut.2025.05.027)
Supplement: multimedia component 1 [file mmc1.docx]

**Supplemental Methods 1.**

The mathematical expression for VanderWeele’s 4-way decomposition is shown in Equation 1 (1). In our study, we conduct the decomposition on the ratio scale. However, for simplicity, it is shown here on the difference scale: Y denotes the outcome, and M is the potential mediator, which is binary (present=1 and absent=0). The binary exposure is indicated by present =1 and absent =0. 1Y_1_ – Y_0_ is the effect of the exposure on the outcome (the total effect (TE)); M_1_ – M_0_ is the effect of the exposure on the mediator; Y_1M0_ is the outcome when the exposure is present, but the mediator is not; and Y_0M0_ is the outcome when neither the exposure nor mediator is present. The first component of Equation 1 corresponds to the controlled direct effect (CDE) when the mediator is set to 0 (i.e., counterfactual scenario when no one is participating in the interventions), the second component is the reference interaction (INTref), the third component is the mediated interaction (INTmed), and the fourth component the pure indirect effect (PIE).

Equation 1

$Y_{1}-Y_{0}=\left( Y_{1M0}-Y_{0M0} \right)+\left( Y_{1M1}-Y_{1M0}-Y_{0M1}+Y_{0 M0} \right)\left( M_{0} \right)+\left( Y_{1 M1}-Y_{1M0}-Y_{0M1}+Y_{0M0} \right)\left( M_{1}-M_{0} \right)+\left( Y_{0M1}-Y_{0M0} \right)\left( M_{1}-M_{0} \right)$

Equation 1 can be rearranged to demonstrate that the difference between the TE and the CDE is equal to the sum of the INTref, INTmed, and PIE (Equation 2).

Equation 2

$Y_{1}-Y_{0}-\left( Y_{1M0}-Y_{0M0} \right) = \left( Y_{1M1}-Y_{1M0}-Y_{0M1}+Y_{0 M0} \right)\left( M_{0} \right)+\left( Y_{1 M1}-Y_{1M0}-Y_{0M1}+Y_{0M0} \right)\left( M_{1}-M_{0} \right)+\left( Y_{0M1}-Y_{0M0} \right)\left( M_{1}-M_{0} \right)$

**Reference**

1. Vanderweele TJ. A unification of mediation and interaction: A 4-way decomposition. Epidemiology. 2016 Sep;27(5):e36. doi: 10.1097/EDE.0000000000000527.

**
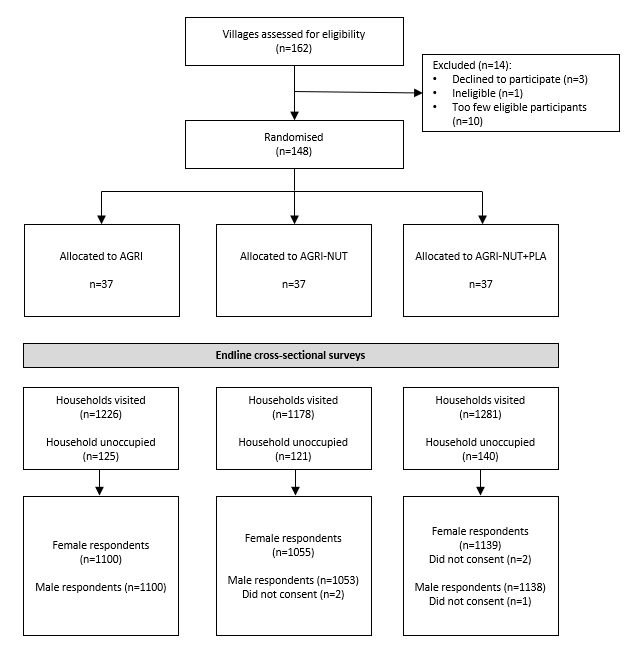
**

**Supplemental Figure 1.** Flow chart of study participants.

Notes: AGRI & AGRI-NUT=Interventions with women’s groups using participatory videos on nutrition-sensitive agriculture and nutrition-specific topics; AGRI-NUT+PLA=Same as AGRI & AGRI-NUT, with the addition of nutrition-specific Participatory Learning and Action meetings.


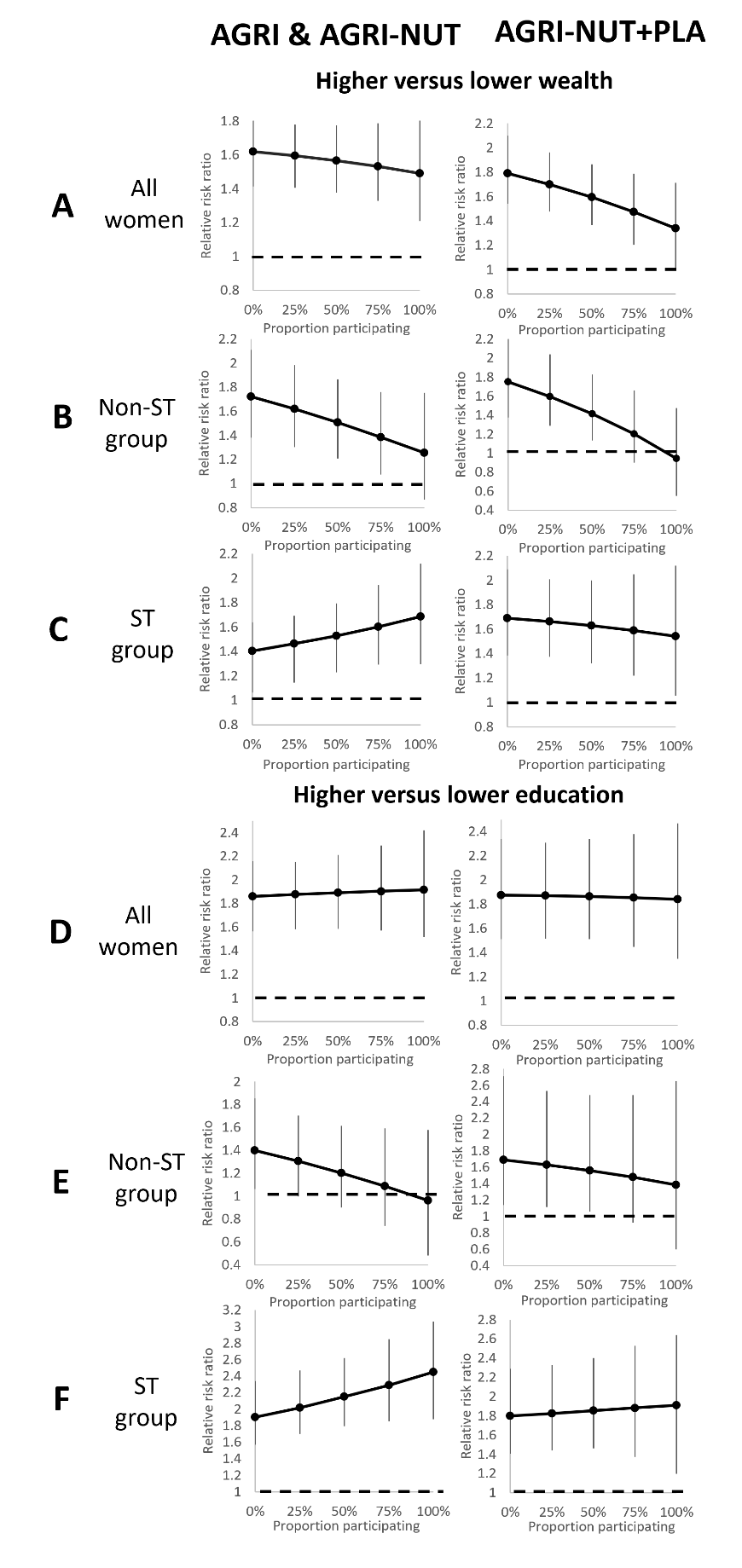


**Supplemental Figure 2.** Wealth and educational inequalities in MDD-W among all women and within non-ST/ST groups across different counterfactual scenarios of participation in AGRI & AGRI-NUT (left panels) and AGRI-NUT+PLA (right panels) interventions.

Notes: Results from 4-way decomposition analyses for the controlled direct effect calculated with intervention participation fixed to 0%, 25%, 50%, 75%, and 100%. Error bars are 95% confidence intervals calculated from bootstrapped standard errors (1000 replications).

AGRI & AGRI-NUT=Interventions with women’s groups using participatory videos on nutrition-sensitive agriculture and nutrition-specific topics; AGRI-NUT+PLA=Same as AGRI & AGRI-NUT, with the addition of nutrition-specific Participatory Learning and Action meetings. MDD-W=minimum dietary diversity for women; non-ST=women not from Scheduled Tribes; ST=Scheduled Tribe. Higher and lower education is defined as women with ≥5 or <5 years of schooling and higher and lower wealth as being in the top or bottom 50% of a wealth score derived as the first principal component from a Principal Component Analysis of ownership of 16 household assets.


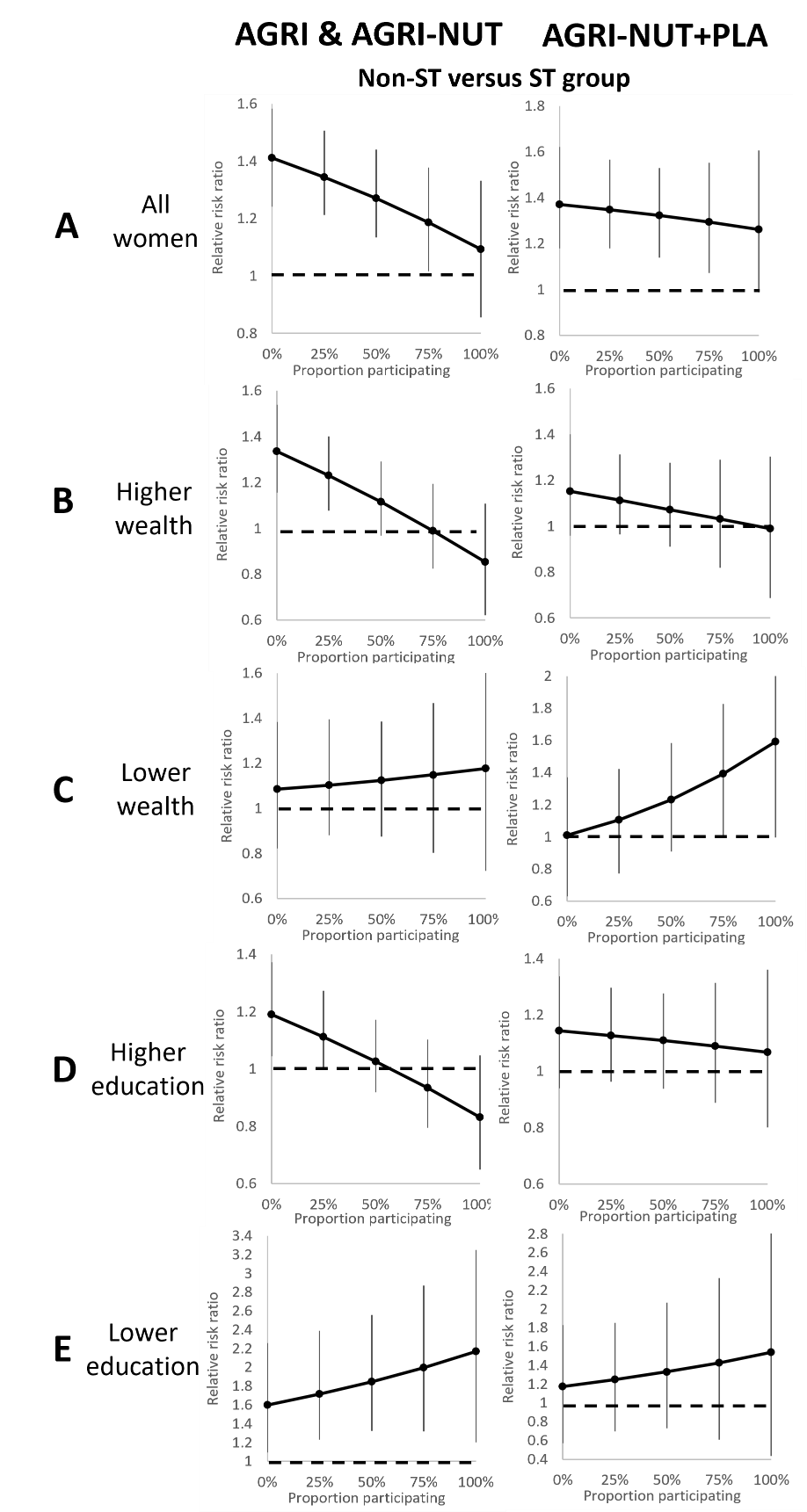


**Supplemental Figure 3.** Non-ST/ST inequalities in MDD-W among all women and within wealth and education groups across different counterfactual scenarios of participation in AGRI & AGRI-NUT (left panels) and AGRI-NUT+PLA (right panels) interventions.

Notes: Results from 4-way decomposition analyses for the controlled direct effect calculated with intervention participation fixed to 0%, 25%, 50%, 75%, and 100%. Error bars are 95% confidence intervals calculated from bootstrapped standard errors (1000 replications).

AGRI & AGRI-NUT=Interventions with women’s groups using participatory videos on nutrition-sensitive agriculture and nutrition-specific topics; AGRI-NUT+PLA=Same as AGRI & AGRI-NUT, with the addition of nutrition-specific Participatory Learning and Action meetings. MDD-W=minimum dietary diversity for women; non-ST=women not from Scheduled Tribes; ST=Scheduled Tribe. Higher and lower education is defined as women with ≥5 or <5 years of schooling and higher and lower wealth as being in the top or bottom 50% of a wealth score derived as the first principal component from a Principal Component Analysis of ownership of 16 household assets.

**
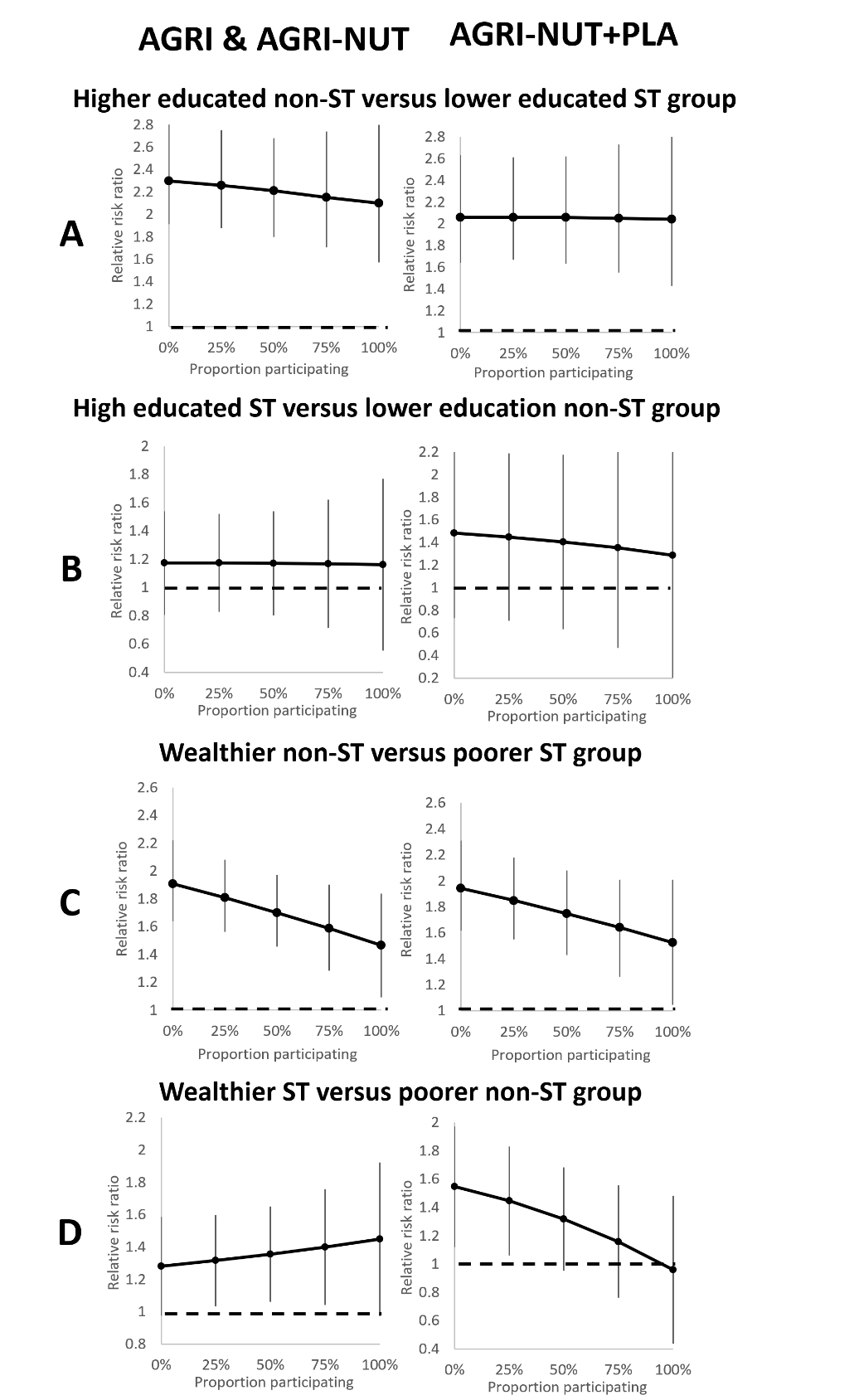
**

**Supplemental Figure 4.** Non-ST/ST and wealth, and non-ST/ST and educational inequalities in MDD-W across different counterfactual scenarios of participation in AGRI & AGRI-NUT (left panels) and AGRI-NUT+PLA (right panels) interventions.

Notes: Results from 4-way decomposition analyses for the controlled direct effect calculated with intervention participation fixed to 0%, 25%, 50%, 75%, and 100%. Error bars are 95% confidence intervals calculated from bootstrapped standard errors (1000 replications).

AGRI & AGRI-NUT=Interventions with women’s groups using participatory videos on nutrition-sensitive agriculture and nutrition-specific topics; AGRI-NUT+PLA=Same as AGRI & AGRI-NUT, with the addition of nutrition-specific Participatory Learning and Action meetings. MDD-W=minimum dietary diversity for women; non-ST=women not from Scheduled Tribes; ST=Scheduled Tribe. Higher and lower education is defined as women with ≥5 or <5 years of schooling and higher and lower wealth as being in the top or bottom 50% of a wealth score derived as the first principal component from a Principal Component Analysis of ownership of 16 household assets.
